# Risk factors for ureteroenteric stricture after radical cystectomy and urinary diversion: A systematic review

**DOI:** 10.1080/2090598X.2023.2239107

**Published:** 2023-07-23

**Authors:** Osama Mahmoud, Ulrich Krafft, Mulham Al-Nader, Jochen Heß, Claudia Kesch, Mostafa AbdelRazek, Ahmad Abolyosr, Gamal A Alsagheer, Omar Mohamed, Atef Fathi, Boris A. Hadaschik, Stephan Tschirdewahn

**Affiliations:** a Department of Urology, University Hospital Essen, Essen, Germany; b Department of Urology, Qena Faculty of Medicine, South Valley University, Qena, Egypt

**Keywords:** Bladder cancer, radical cystectomy, urinary diversion, ureteroenteric stricture

## Abstract

**Introduction:**

Ureteroenteric stricture (UES) is the leading cause of renal function deterioration after radical cystectomy (RC) and urinary diversion (UD). The aim of the present review is to summarize studies that discussed the risk factors associated with UES development. Identifying the responsible factors is of importance to help surgeons to modify their treatment or follow-up strategies to reduce this serious complication.

**Materials and Methods:**

A comprehensive search of the literature using the PubMed database was conducted. The target of the search was only studies that primarily aimed to identify risk factors of UES after RC and UD. References of searched papers were also checked for potential inclusion.

**Results:**

The search originally yielded a total of 1357 articles, of which only 15 met our inclusion criteria, comprising 13, 481 patients. All the studies were observational, and retrospective published between 2013 and 2022. The natural history of UES and the reported risk factors varied widely across the studies. In 13 studies, a significant association between some risk factors and UES development was demonstrated. High body mass index (BMI) was the most frequently reported stricture risk factor, followed by perioperative urinary tract infection (UTI), robotic-assisted radical cystectomy (RARC), occurrence of post-operative Clavian grade ≥ 3 complications and urinary leakage. Otherwise, many other risk factors were reported only once.

**Conclusion:**

The literature is still lacking well-designed prospective studies investigating predisposing factors of UES. The available data suggest that the high BMI, RARC and complicated postoperative course are the main risk factors for stricture formation.

## Introduction

Despite major advances in health care and the availability of well-trained surgeons around the world, radical cystectomy (RC) remains one of the most risky urological procedures, with a high early complication rate of 25% to 64% and a mortality risk of up to 5.7%. In addition, up to 50% of patients still develop complications years later, mainly related to urinary diversion (UD) [1–5].

The most serious long-term consequence of UD is renal function deterioration, which affects 20–35% of patients [6,7]. Many factors are related to renal impairment including patient’s age, chronic hypertension, diabetes, baseline renal function and diversion-related factors. The group from Bern reported a deterioration of renal function after 10 years in 36% of patients with ileal conduit and 21% of patients with neobladder. They found that urinary tract obstruction at any level is an independent predictor of renal function deterioration [7]. Gilbert et al reviewed data of 1,565 patients who underwent different forms of diversion, Kaplan–Meier analysis at 5 years showed 16% incidence of renal impairment/failure. An ureteroenteric stricture (UES) occurred in 13%, and it was the leading cause of renal function changes [8]. Eisenberg et al analyzed changes in renal function in 1.631 patients, after 10 years follow-up; they found a decrease in renal function in most patients. In the multivariate analysis, risk factors contributing to renal function decline were patient age, preoperative renal function, chronic hypertension, postoperative hydronephrosis, pyelonephritis and the most important factor was UES (HR 1.6, *p* < 0.0001) [9].

To date, there is no consensus in the literature on factors associated with the development of UES, despite the presence of many large studies. This can be attributed to the retrospective nature of these studies or the study design itself; some studies were either primarily based on assessing the influence of one factor on the development of the stricture, e.g. the type of ureteroileal anastomosis or preoperative radiotherapy (RT), or they did not include all possible contributing pre-operative and postoperative factors [10–26]

We tried in this systematic review to define the reported risk factors of UES in the literature, we focused only on studies that identified these factors through multivariate analysis including many possible contributing factors.

## Materials and methods

### Search strategy

A comprehensive search of the PubMed database has been conducted to identify studies that address the risk factors associated with the development of UES. The systematic review was performed according to the Preferred Reporting Items for Systematic Reviews and Meta-Analyses (PRISMA) checklist [27]. The computer search was conducted in December 2022 to find relevant studies that were published between 1966 and 2022. The search was performed using different combinations of the following MeSH terms: ‘cancer, bladder’, ‘cystectomy’, ‘cystectomy/adverse effects’, ‘urinary diversion’, ‘urinary diversion/adverse effects’, ‘ureter’, ‘ureteral obstruction’, ‘anastomosis, surgical/adverse effects’ and ‘anastomosis, surgical’.

The titles were screened to identify the relevant articles. Results were deduplicated using EndNote program. For the relevant titles, the abstracts and then the articles were inspected for our inclusion criteria. The list of references in the finally selected papers were also evaluated for potentially relevant studies. We included only papers that investigated the risk factors of UES after RC and UD using multivariate analysis across many possible contributing factors. Articles that studied the effect of one variable, e.g. the technique of anastomosis without adjusting it with the other possible contributing factors were excluded. Only papers that were written in English were included.

### Data extraction

The following information were extracted from each eligible study: publication details (title, first author and publication year), number of the patients, operative parameters (approach, type of diversion and technique for ureteroenteric anastomosis), the median follow-up, natural history of UES (incidence, laterality of UES and mean time to diagnosis) and the independent predictors of UES development in the multivariate analysis, which are considered the main outcome of the review.

## Results

**Figure 1.**
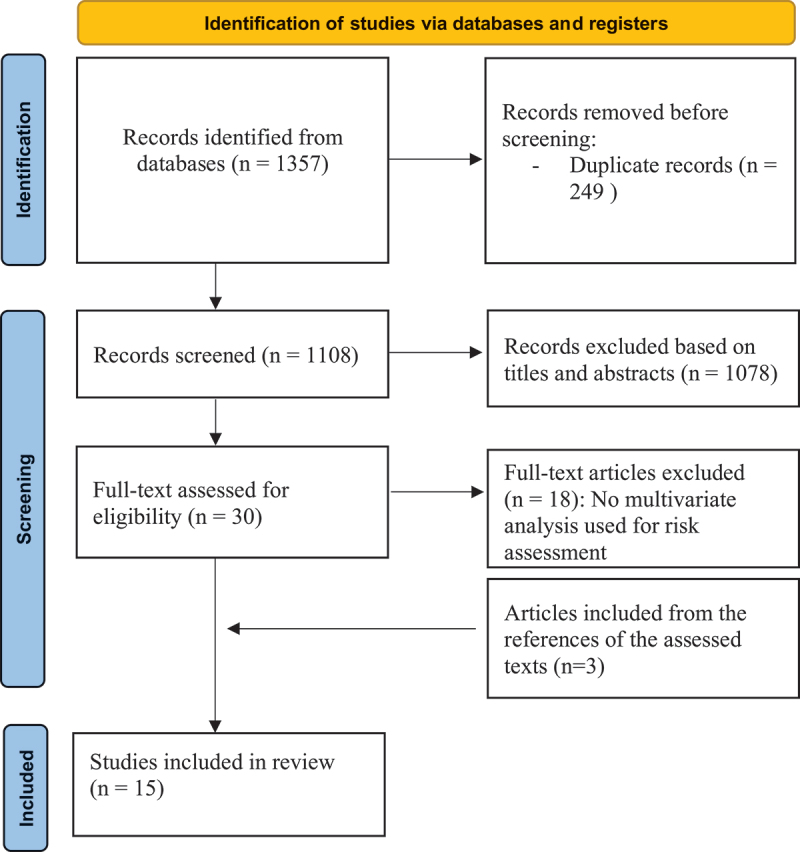
PRISMA flow diagram.

The PRISMA flowchart is shown in Figure 1. The search using the previously mentioned mesh phrases yielded 1357 articles in total. After the exclusion of duplicated and non-relevant ones based on the titles, 45 were selected for possible inclusion. The abstracts of these studies were reviewed, which results in exclusion of another 15 abstracts. After reviewing the manuscripts of the selected abstracts, only 12 articles were found to fit our inclusion criteria. Another three articles were found to be eligible through reviewing the references in the previously red manuscripts. Finally, 15 were included in the analysis.

Table 1 provides detailed information on the study characteristics and the results of multivariate analysis [12–26]. The included studies were observational, retrospective and published between 2013 and 2022. Of the 15 articles, 6 were open radical cystectomy (ORC) series, 7 contained both open and robotic-assisted (RARC) and 2 were RARC only. The sample size in the studies varied between 135 and 2,888 and included a total of 13, 481 patients. The type of ureteroenteric anastomosis was mentioned in 12 articles, Bricker was the predominant choice (*n* = 7), followed by Wallace (*n* = 1) and both techniques were used in four studies. The median follow-up differed widely between the studies, ranging from 8.2 months to 12.4 years. There was a wide discrepancy in the stricture rates among the studies (2.6–17%). The median time to stricture diagnosis was similar across the studies lying between 3 and 10 months, only one study reported a later stricture diagnosis at the median time of 15.8 months [13]. Left-sided ureteral stricture was the most predominant (42.2–66%), followed by right-sided stricture (9.5–44.7%) and bilateral involvement (5–42%). Patient presentation was reported in only six articles, 70–98% of patients were presented with symptoms in four studies, while in two studies most patients were asymptomatic and were diagnosed during routine follow-up.

**Table 1.** Studies discussed risk factors associated with ureteroenteric stricture development.

Authors, year	Patients No.	Type of diversion	Technique of ureteroenteric anastomosis	UES Incidence	Median Follow-up	Median time to diagnosis	Affected side	Presentation	Risk factors for stricture
Liu et al. 2022 [22]	180 -ORC:75 -RARC:105	IC and ONB	Bricker and Wallace	12.2%	NR	11 mo.	Left: 45.4% Right: 40.9% Bilateral: 13.6%	NR	Blood transfusion (HR 0.144, 95% CI: 0.046–0.451; *P* = 0.001) Extracorporeal diversion (HR 3.39, 95%CI: 1.24–9.28, *P* = 0.017) Postoperative UTI (HR 3.62, 95% CI: 1.4–9.3,*P* = 0.007)
Krafft et al. 2021 [23]	135	IC, ONB and CCD	Bricker and Wallace	15.5%	14 mo.	3 mo.	Left: 47.6% Right: 9.5% Bilateral: 42%	NR	Preoperative chemotherapy (OR 9.7, 95%CI:2–46.2; *P* = 0.004) Clavien ≥3 complication (OR 4, 95%CI: 1.36–11.82, *P* = 0.012)
Reesink et al. 2021 [24]	279 -ORC:192 -RARC (ICUD):87	IC and ONB	Bricker	17% -ORC: 13% -RARC:25.3%	50 mo.	3 mo.	Left: 48.9% Right: 21.3% Bilateral: 29.8%	Symptomatic (40.4%)	RARC (HR 2.36, 95% CI: 1.32–4.2; *P* = 0.004)
Faraj et al. 2021 [25]	573 -ORC:337 -RARC (ICUD):39 -RARC (ECUD):197	IC and ONB	Bricker and Wallace	8.2% -ORC:8% -RARC (ICUD):1.6% -RARC (ECUD):9.6%	ORC:55 mo. RARC (ICUD):71 mo. RARC (ECUD):70 mo.	5 mo.	Left: 51.1% Right: 44.7% Bilateral: 4.3%	Symptomatic (98%)	BMI (HR 1.07, 95% CI: 1.02–1.13; *P* = 0.003).
Ericson et al. 2020 [26]	968 -ORC:279 -RARC (ICUD):307 -RARC (ECUD):382	IC, ONB and CCD	Bricker	11.3% -ORC:9.3% -RARC (ICUD):13% -RARC (ECUD):11.3%	ORC:22 mo. RARC (ICUD):16.3 mo. RARC (ECUD):10.6 mo.	4.67 mo.	Left: 50.5% Right: 31.2% Bilateral: 17.4%	NR	RARC (ICUD) (HR 1.66, 95% CI: 1–2.74,*p* = 0.05) BMI (HR 1.05, 95% CI: 1.02–1.09; *P* < 0.01).
Yang et al, 2020 [12]	2.285	IC and ONB	Bricker and Wallace	8%	10.7 years	7 mo.	Left: 53% Right: 28% Bilateral: 18%	Symptomatic (77%)	BMI (OR 1.06, 95% CI: 1.02–1.09;*P* = 0.0009). Clavien ≥3 complication (OR 2.85, 95%CI: 1.90–4.28; *P* < 0.0001)
Benson et al, 2020 [13]	418	ONB	NR	8.9%	Mean: 57 mo.	Mean time: 15.8 mo.	Left: 65% Right: 27% Bilateral: 14%	NR	Perioperative UTI (HR 2.4, 95% CI 1.09–5.09, *p* = 0.03) Recurrent UTI (HR 5.1, 95% CI 2.4–11,*p* < 0.001) History of RT (HR 11.5, 95% CI 2.5–53.4,*p* = 0.002)
Goh et al, 2019 [14]	1781 -ORC:1449 -RARC:332	Continent and incontinent UD	NR	ORC: 8.3% RARC: 13.9%	At 2 years	NR	NR	NR	RARC (HR 1.64, 95%CI 1.23–2.19) Preoperative hydronephrosis (HR 1.51, 95%CI 1.17–1.94)
Hosseini et al, 2018 [15]	371 (RARC +ICUD)	IC and ONB	Wallace	6.5%	33 mo.	165 d.	Left: 63% Right: 29% Bilateral: 8%	NR	Urinary leak (HR 5.17, 95% CI 1.85–14.41,*p* = .002)
Katherine et al, 2018 [16]	2.888	Continent and incontinent UD	NR	4.2%	32 mo.	NR (80% within 1^st^ 3 years)	Left: 53.6% Right: 40.6% Bilateral: 5.7%	Symptomatic (76.4%)	BMI (HR 1.04, 95% CI 1.01–107, *p* < 0.0001) Male gender (HR 1.81, 95% CI 1.13–2.91,*p* = 0.014) Prior abdominal surgery (HR 3.40, 95%CI 1.84–6.28, *p* = 0.013) ASA III/IV (HR 1.88, 95% CI 1.2–2.9, *p* = 0.005)
Ahmed et al, 2017 [17]	440 (RARC)	IC	Bricker	13%	17 mo.	5 mo.	Left: 45% Right: 29% Bilateral: 25%	Symptomatic (25%)	BMI (OR 1.07, 95% CI 1.01–1.13, *p* = 0.02) ICUD (OR 3.28, 95% CI 1.41–7.61, *p* = 0.006) UTI (OR 2.68, 95% CI 1.31–5.49, *p* = 0.007) Urinary leak (OR 3.85, 95% CI 1.05–14.1,*p* = 0.04) Length of the right resected ureter (OR 0.66, 95% CI 0.50–0.88, *p* = 0.004) ≥ 30 d eGFR (OR 0.85, 95%CI 0.74–0.98,*p* = 0.03)
Shah et al, 2015 [18]	1964	IC, ONB and CCD	Bricker	2.6%	12.4 years	10 mo.	Left: 66% Right: 29% Bilateral: 5%	Symptomatic (70%)	No independent predictor for stricture
Richards, 2014 [19]	463 -ORC: 439 -RARC: 24	IC and ONB	Bricker	12.5%	459 d.	Right ureter: 235 d. Left ureter: 232 d.	Left: 56.9% Right: 24.1% Bilateral: 19%	NR	Clavien ≥3 complication (HR2.11,1.01–4.40) Urine leak (HR 3.37, 1.08–10.46)
Anderson et al, 2013 [20]	478 -ORC:375 -RARC:103	IC, ONB and CCD	Bricker	ORC: 8.5% RARC: 12.6%	8.2 mo.	5.3 mo.	Left: 42.2% Right: 24.4% Bilateral: 33.3%	NR	No independent predictor for stricture
Large et al, 2013 [21]	258	IC and ONB	Bricker	16.6%	-Running sututres gp: 351 d. -Interrupted sututresgp: 497 d.	-Running sututres gp: 289 d. -Interrupted sututres gp.: 213 days	Left: 46.5% Right: 34.8% Bilateral: 18.6%	NR	Postoperative UTI (HR 2.4, 95% CI 1.2–5.1, *p* = 0.02) Running technique (HR 1.9, 95% CI 1.0–3.7,*p* = 0.05)

IC= Ileal conduit; ONB=Orthotopic neobladder; BMI=Body mass index; ORC=open radical cystectomy; RARC=Robotic-assisted radical cystectomy; UD=Urinary diversion; ICUD=Intracorporeal urinary diversion; UTI=Urinary tract infection; RT=Radiotherapy; NR=Not reported.

Of 15 articles, a significant relationship between many factors and UES was demonstrated in 13 studies, on the other hand, no correlation between patient or disease factors specific and the development of UES could be found in two articles. The most frequently mentioned stricture risk factor was BMI that was reported in five articles, followed by postoperative perioperative urinary tract infection (UTI) in four articles. The occurrence of major postoperative complications (Clavien-Dindo ≥3), urinary leakage and RARC each of which was reported three times. In two studies intracorporeal UD (ICUD) with associated with higher stricture risk adjusted to extracorporeal UD (ECUD). Otherwise, the link between many other factors and UES was observed only one time: these factors were either related to patient characteristics including the male gender, ASA score III-IV, prior abdominal surgery, history of RT, history of chemotherapy, preoperative hydronephrosis, postoperative renal function and node positive disease or related to the surgery including (ECUD), the use running sutures in creating the ureteroileal anastomosis the length of resected ureter, and blood transfusion (Table 1).

## Discussion

### Demographic and clinicopathological factors

The patient demographic and clinical features are important determinants for the perioperative outcome of RC and UD [28,29]. These factors are static and cannot be changed. However, their identification is important to characterize high-risk patients who should be treated in high-volume centers with experienced surgeons. Increasing patient age and comorbidities were associated with early postoperative complications in many studies, in addition, their negative influence on the long-term renal function is known [9,29]. None of the published studies showed an association between age and UES development; nevertheless, the poor physical condition of the patient, represented by an increase in the ASA (American Society of Anesthesiologists) score, was associated with two times stricture risk in one study [16]. In the same study, Katherine et al. found a link between male gender and UES, however, this association may be influenced by the high proportion of men in the study, which represents 75% of their cohort [16].

It is obvious that the high BMI is a main risk factor for UES among the patient characteristics. Five studies in our review reported the associated hazardous effect [12,16,17,25,26]. Moreover, some studies that did not observe this association either consisted of a relatively small sample size [15,21] or did not include the BMI in the analysis at all [14]. The left ureter is usually brought to the right side under the sigmoid mesentery before the ureteroenteric anastomosis; therefore, enough ureteral length is required, which is difficult to achieve in obese patients due to high abdominal obesity. Consequently, the left ureter may be exposed to extensive dissection or tension during anastomosis, factors which increase the incidence of UES. This explains also the well-known predominance of left-sided stricture. In addition to previous surgical problems, the healing process in obese patients is not optimal and impaired by associated morbidities (e.g. diabetes mellitus) or some mediators that detach from the mesenteric fat tissue [30].

Other factors that increase surgical difficulty and predispose to intra- and posoperative complications include previous exposure to major abdominal surgery and RT. In the largest series in our review: the risk of developing a stricture within 10 years was 1.9% in patients without prior abdominal surgery compared to 9.3% in patients with prior surgery [16]. Prior abdominal surgery is associated with adhesions, which in turn distorts the tissue planes and consequently predisposes to high incidence of perioperative complications like anastomotic leak and abdominal infections, factors which increase the risk of inflammatory stricture. Also, the distortion of the tissue planes increases the possibility of ureteral devascularization during dissection and subsequently the incidence of ischemic stricture. Radiation induces tissue ischemia and fibrosis, which could affect the healing process of the ureteral anastomosis. This theory seemed logical, but the data in the literature are still contradictory. Bensen et al found that preoperative RT is associated nearly with 11 times higher risk for stricture formation [13]; however, only 2% of the patients received the preoperative therapy, which is small proportion that could affects the results. Yang et al, examined 2,285 patients, with 12.5% of patients receiving RT, which is considered an appropriate number; in the univariate analysis, RT was not a risk factor (OR 1.25, CI: 0.80–1.87, *p* = 0.35) [12]. Despite the lack of clear evidence from the literature, surgeons should be careful and try to select a viable ileal and ureteral segment to be used for diversion outside the irradiation field or to use directly a transverse colonic segment for diversion.

Patients undergoing UD can present with unilateral or bilateral hydronephrosis due to tumor obstruction or a defunctionalized bladder. Preoperative hydronephrosis is an independent predictor of UES in one study [14]. This could be explained by the pathological changes that occur in the ureteral wall following long-standing obstruction and the associated toxic effect of recurrent infection. These changes could impair ureteral elasticity and the healing process. Hautmann et al published an important report that is not included in our review, as the hydronephrosis was not adjusted to the other possible confounding factors. The authors reviewed the data of 953 patients with neobladder. They reported 19.3% stricture rate at 10 years in patients with preoperatively obstructed ureters versus only 6.4% for patients with non-dilated ureters [10].

Only one study showed a correlation between pathological features and UES development: patients with node positive disease were more likely to develop stricture. The authors explained that neovascularization associated with nodal metastasis can alter the blood supply to the ureters and lead to scarring. Another explanation, which was not investigated in the study, is that aggressive disease and lymphadenopathy increase the difficulty of ureteral dissection and the possibility of devascularization [16].

### Surgery related factors

Both ORC and RARC are comparable in the literature with regard to the oncological outcome and perioperative surgical complications [31]. Whether the surgical approach influences the long-term functional outcome or not is still questionable. Some urologists suggested that RARC would be associated with higher incidence of UES. Seven studies in our review have included both open and RARC cohorts, three of them showed high stricture rate among in the robotic group [14,24,26]. This higher stricture rate in RARC may be related to the inclusion of cases operated on early in the surgeon’s experience, as the lack of tactile sense and higher magnification may increase devascularization of the ureter. For example, Goh et al. found that a high hospital volume in the adjusted analysis was protective against stricture, which means that with increasing experience of the surgeon the result would be comparable (Goh et al., 2020). A promising technique to reduce the incidence of UES is the use of indocyanine green (ICG) with near-infrared fluorescence to identify ureteral blood supply during distal ureterectomy. Ahmadi et al used ICG in 47 patients and compared them with 132 patients without ICG. The length of excised ureter was significantly higher in the ICG group than in the other group, and no UES developed in the ICG group at one year compared to 10% in the non-ICG group (*p* = 0.020) [32]. Long-term follow-up studies comparing both open and robotic techniques and evaluating the effects of using near-infrared fluorescence are still needed.

The best technique for ureteroenteric anastomosis has been a question in many studies over the last 30 years, currently there is consensus that antireflux anastomosis is associated with a higher incidence of UES without having a positive effect on long-term renal function [5,33]. With respect to the refluxing techniques, the only published meta-analysis showed a comparable stricture rate between Bricker (2.9%) and Wallace (1.9%) (*p* = 0.57) [34]. In the present review, the effect of the technique on the outcome of the anastomosis was not proved. Regardless of the technique, anastomosis can be performed either with running or interrupted sutures, this was investigated in two studies of our search: Large et al. evaluated 258 patients who underwent various diversions. The interrupted anastomosis was performed with 4–0 polyglactin sutures in 149 patients and with the same suture in 109 patients with running anastomosis. No significant difference in surgical time was found between the two groups. In the multivariate analysis, the running suture technique was associated with stricture formation [21]. In a later study with a larger number of patients and longer follow-up time, the running suture technique did not appear to affect the incidence of UES [19].

The distal end of the ureter is usually compromised during dissection and should be resected. Surgeons want also to adjust the length to avoid redundancy of the ureter or anastomosis under tension. Two studies have investigated whether the length of the resected ureter affects the outcome of anastomosis or not. Richards et al added the length of the ureter in cystectomy specimens plus the separately resected distal ureter in 463 patients of whom 12.5% developed UES. They found no significant correlation between resected length and stricture formation [19]. In contrast, the other study demonstrated the presence of this association. The study included 440 patients undergoing RARC and 13% had UES. The stricture group had a significantly shorter length of resected ureters (left side 15 vs. 20 mm, *p* = 0.02, and right side 15 vs. 22, *p* < 0.001). In multivariate analysis, the association was significant only on the right side. Finally, the authors concluded that further studies are needed as the length of resected ureter only not enough due to the variations between the patients as regard their height and the original ureteral length [17].

In most of the included studies, ureteral stents were routinely used for a period of 1 to 2 weeks postoperatively, whereas three studies did not mention whether or not diversions were stented. Therefore, the effect of stent use on the outcome of ureteroenteric anastomosis was not examined in the analysis of these studies. Few studies compared stricture rates in stented and nonstented patients, and the results were summarized in a recently published meta-analysis. There was no statistically significant difference in the rate of urinary leakage or development of strictures between the two groups. Future prospective randomized studies on this topic are still needed [35].

## 3 Postoperative complications

Some early postoperative complications may predispose to UES development, such as urinary or intestinal leakage, abdominal abscess and UTI. These factors can trigger an inflammatory process at the anastomotic site and predispose to scar and UES formation. The occurrence of Clavian grade complication ≥ 3 was associated with a three times higher risk of stricture formation in one study and four times in another one [12,23], while in one study it was a predictor for stricture on the left side only [19]. Katherine et al in the largest study observed also this relationship, but only in the univariate analysis [16]. Urinary leakage alone was seen to be an independent predictor for UES in three studies. The first was conducted by Richards et al. 2014 in patients undergoing ORC and UD. All anastomoses were Bricker, stented and the anastomoses were tested for water tightness. Twenty percent of patients who developed later right UES had a history of urinary leakage postoperative vs. 6.3% in those free from strictures (*p* = 0.01). In the adjusted analysis, urinary leak was associated with an almost triple risk of developing a right-sided stricture [19]. This significant association was demonstrated also in two robotic series with ICUD [15,17]. The authors of both studies recommended great efforts to ensure adequate watertight anastomosis to minimize the risk of postoperative urinary leakage. Early occurrence of UTI had an almost three times higher risk of stricture in four studies [13,15,17,22]. In addition, Hosseini et al. found that recurrent UTI over a long period of time carry a risk of developing a stricture twice as high as one attack of perioperative UTI, but the authors could not determine if the recurrent UTIs are possible cause of stricture or are the results of pre-existing UES [15].

## Conclusion

Although risk factors of UES development have been discussed in many studies, the exact cause is still unclear. This is due to the presence of some non-measurable factors, mainly surgical and technical that could directly influence the outcome of the anastomosis. Ischemic strictures are the most common form resulting from excessive dissection or RT. Great efforts should be made to maintain the blood supply to the ureter by gentle handling, avoiding unnecessary dissections and minimizing the use of electrocautery. Obese patients are considered a high-risk group for ischemic stricture and should be operated on by experienced surgeons. Inflammatory strictures are less common and resulted mainly from a complicated postoperative course. Special attention should be paid to meticulous surgical technique to avoid urinary and intestinal leakage. Wide spatulation of the ureter, mucosal-to-mucosal suturing and watertight stented anastomosis are basic principles for minimizing the urinary leakage and stricture. Preoperative UTI in patients undergoing UD should be treated, careful follow-up, early detection and treating new episodes of UTI are mandatory in the early postoperative course.
